# Preparation and Characterization of Biomimetic Hydroxyapatite Nanocrystals by Using Partially Hydrolyzed Keratin as Template Agent

**DOI:** 10.3390/nano9020241

**Published:** 2019-02-11

**Authors:** Chunxia Gao, Ke Zhao, Liwei Lin, Jinyu Wang, Yang Liu, Peizhi Zhu

**Affiliations:** School of Chemistry and Chemical Engineering, Yangzhou University, Jiangsu 225009, China; cxgao@yzu.edu.cn (C.G.); m160292@yzu.edu.cn (K.Z.); linlwei@sohu.com (L.L.); jinywang2@gmail.com (J.W.); liuyangyzu@sohu.com (Y.L.)

**Keywords:** hydroxyapatite (HA), nanocrystal, hydrolyzed keratin, fish scales

## Abstract

Hydroxyapatite (HA), a typical inorganic component of bone, is a widely utilized biomaterial for bone tissue repair and regeneration due to its excellent properties. Inspired by the recent findings on the important roles of protein in biomineralization and natural structure of fish scales, keratin was chosen as a template for modulating the assembly of HA nanocrystals. A series of HA nanocrystals with different sizes were synthesized by adjusting the concentration of partially hydrolyzed keratin. The structure and compositions of the prepared HA were characterized by Fourier transform infrared (FTIR) spectroscopy, X-ray diffraction (XRD), Raman spectrum, and Transmission electron microscopy (TEM). Results revealed that the size of the synthesized HA nanocrystals can be controlled by adjusting the concentration of partially hydrolyzed keratin. Specifically, the size of synthesized HA decreased from 63 ± 1.5 nm to 27 ± 0.9 nm with the increasing concentration of partially hydrolyzed keratin from 0 to 0.6g. In addition, in vitro cytocompatibility of synthesized HA nanocrystals were evaluated using the MG-63 cells.

## 1. Introduction

Hydroxyapatite (HA), with chemical composition Ca_10_(PO_4_)_6_(OH)_2_, has been increasingly used in clinical applications, such as orthopedics and dentistry, due to its similarity in crystal structures and compositions to biological apatite that can be found in hard tissues such as teeth and bones [1,2]. Various HA with different crystal structure and morphology have been developed [3]. Among these synthesized HA materials, nanostructured HA has received much attention due to their higher specific surface areas that enhance the adhesion of cells, proteins, and drugs [4]. In addition, inspired by the process of biomineralization of natural bones, various macromolecules have been chosen as a templating agent to manipulate the growth of HA crystals [5]. During the mineralization process of HA crystals, macromolecules can play the predominant role in the nucleation, growth, orientation, and structure of HA crystals [6]. Among these templating agents, proteins have received much attention due to their functional groups on side chains which can chelate Ca^2+^ ions and form hydrogen bonding with PO_4_^3−^ and H_2_O on the surface of the minerals [7,8]. 

As one type of protein, keratins widely exist in biological systems, comprising the main component of natural materials such as hair, fur, wool, nails, hooves, and fish scales [9,10,11,12,13,14]. The helical molecular structure and macromolecular organization of α-keratin impart these materials with notable characteristics such as strength and flexibility [15]. The fish scales are composed of calcium-deficient HA and extracellular matrix, mainly keratin, which together form a highly ordered three-dimensional hierarchical structure [16,17,18]. HA derived from the fish scale, have been considered as an ideal scaffold for the use in orthopedics since the minerals Na^+^, Mg^2+^, F^−^, Cl^−^, K^+^, and CO_3_^2−^ are present in HA derived from fish scale and make the apatites structurally similar to the apatites in a natural bone [19,20]. Keratin in fish scales act as one of the templates for the orientation of the mineral phase but how keratin regulates the morphology, size, and crystallinity of the apatite minerals in fish scales remains unknown [21,22,23]. Moreover, inspired by recent findings on the important roles of protein in biomineralization and natural structure of fish scales, we hypothesized that negatively charged segments of keratin could play an important role in regulating the mineralization process of HA crystals. To synthesize HA nanocrystals with controllable morphologies and sizes, keratin was chosen as a template. In the present study, partially hydrolyzed keratin was used due to the insolubility of keratin in an aqueous solution.

The main objective of this study was to investigate the effects of the concentration of partially hydrolyzed keratin on the morphology, size and biological activity of synthesized HA nanocrystals. The role of keratin on regulating the process of mineralization of HA crystals was investigated. Compositions, morphology and crystal structure of synthesized HA nanocrystals were also characterized. In addition, in vitro cytocompatibility of these synthesized HA nanocrystals was evaluated using human osteosarcoma cells (MG-63) regarding their proliferation.

## 2. Materials and Methods 

### 2.1. Materials

The partially hydrolyzed keratin was purchased from Yuanchen Co., Ltd. (Wuhan, China). Ca(NO_3_)_2_·4H_2_O, (NH_4_)_2_HPO_4_ and ammonium hydroxide were purchased from Sinopharm Chemical Reagent Co., Ltd. (Beijing, China). All of the reagents were used as received without treatment or further purification.

### 2.2. Preparation of HA Nanocrystals

In this work, five groups of HA samples were synthesized, and the HA synthesized without keratin was design as S1 and HA derived from carp scale design as S6. S1 and S6 were set as the control groups. The formulae of all groups are shown in Table 1. For HA synthesis, Ca(NO_3_)_2_·4H_2_O and (NH_4_)_2_HPO_4_ were chosen as the precursors of calcium and phosphorus source to synthesize HA by the co-precipitation method [24]. The partially hydrolyzed keratin served as soft templates for the synthesis of HA nanocrystals. Specifically, 9.54% Ca(NO_3_)_2_·4H_2_O was prepared and added a different concentration of partially hydrolyzed keratin (0 g, 0.02 g, 0.1 g, 0.2 g and 0.6 g corresponding to S1, S2, S3, S4, and S5). To get the homogenous solution, the mixture stirred at 95 °C for 2 h. Then 3.2% (NH_4_)_2_HPO_4_ solution was dripped into the heated mixture solution, and pH value of the mixed solution was adjusted at 9.2 by the ammonium hydroxide solution. The suspension needed further 4 h stirring, and then aged at the room temperature for 24 h. Finally, the obtained precipitate was washed by deionized water three times to remove partially hydrolyzed keratin and other soluble substances. Then, the samples were dried by lyophilization (Lyoquest- 55, Telstar, Barcelona, Spain). To prepare the control group, 20 g grass carp scales were dissolved in 100 mL 5M NaOH solution under stirring for 4 h at 95 °C [25]. Next, the insoluble precipitate was collected and washed by deionized water 3 times and dried by lyophilization

### 2.3. Characterization of HA Nanocrystals 

The size and morphology of obtained HA nanoparticles were characterized by transmission electron microscopy (TEM, Tecnai C2 F30 S-Twin, FEI, Hillsboro, OR, USA). Wide-angle X-ray diffraction (XRD, X′ pert-MPD, Philips, Amsterdam, The Netherlands) was used to determine the presence of crystalline phases in these synthesized samples. The XRD analysis was performed using Ni-filtered CuKα radiation (λ = 1.5402 Å) in a step-scan mode (2° per minute) in the 2θ range 10–60°. Composition analysis of the prepared scaffolds was performed using Fourier transform infrared (FT-IR) spectroscopy (ALPHA, BRUKER, Karlsruhe, Germany). FT-IR was performed in the wave-number range of 400–4000 cm^−1^, each FTIR spectrum was obtained from 40 scans at a resolution of 2 cm^−1^. The molecular structure was further analyzed by Raman spectroscopy (DXR, GX-PT-2412, Thermo, Waltham, MA, USA) with 532 nm laser as excitation wavelength.

### 2.4. In Vitro Cytocompatibility 

In vitro cytocompatibility of the prepared samples was evaluated using a Human osteosarcoma cell line MG-63 cells, obtained from the Stem Cell Bank, Chinese Academy of Sciences (Shanghai, China). Prior to seeding with cells, the samples (50 μg/mL) were sterilized using ethylene oxide gas and then placed in 96-well culture plates. MG-63 cells were seeded onto samples with a density of 5000 cells/well (n = 3). Alpha-modified minimum essential medium with L-Glutamine and Phenol Red (α-MEM, Gibco, Grand Island, NY, USA), supplemented with 10% heat-inactivated fetal bovine serum (FBS, GibcoTM, Invitrogen, Grand Island, NY, USA) was used as the culturing medium. A Thermanox^®^ culture plastic plate (TCP) was used as the control group. The cultures were incubated at 37 °C in a humidified atmosphere of 95% air and 5% CO_2_, with the medium changed every 2 days. After the incubation for predetermined days, cell viability was assessed by Live/Dead staining assay and cell proliferation assay. The tests were repeated 3 times for each cement formulation (n = 3). The LIVE/DEAD^®^ Viability/Cytotoxicity Kit (Thermo Fisher Scientific, L-3224, Waltham, MA, USA) was used according to the manufacturer’s instructions. Briefly, after the cell culture medium was removed, 100 μL of PBS containing 4 μM calcein-AM and 2 μM ethidium homodimer-1 was added to stain live and dead cells, respectively. After incubating for 10 min at 37 °C, cells were then examined via epifluorescence microscopy (Olympus ix53, Hataya, Japan). For each sample, five images were taken.

After incubation for 1, 3 and 5 days, cell proliferation was examined using Cell Counting Kit-8 (CCK-8, Dojindo, Kumamoto, Japan), in accordance with the manufacturer’s instruction. Briefly, samples were removed after each incubation, rinsed two times with PBS and added 100 μL PBS. Then, 10 μL of CCK-8 reagent was added to each sample and cultured in an incubator at 37 °C with 5% CO_2_ for an additional 2 h. After 2 h incubation, 100 μL of media were transferred to each well of a 96-well plate to measure absorption value at a wavelength of 450 nm using a microplate reader (Elx-800, bio-Tek instruments, Winooski, VT, USA).

### 2.5. Statistical Analysis

The numeric data were expressed as mean ± standard deviation (SD) and analyzed using one-way analysis of variance (ANOVA).

## 3. Results and Discussion

**Figure 1:**
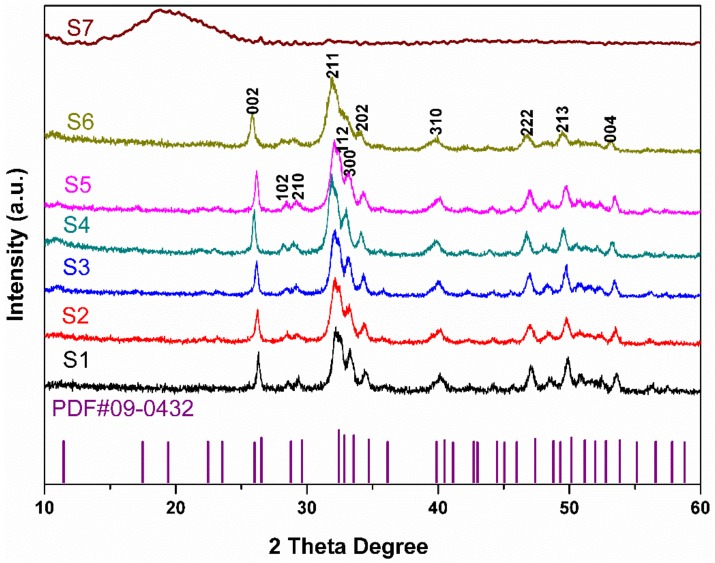
XRD patterns of synthesized HA samples (S1–S5), HA derived from carp scales (S6) and partially hydrolyzed keratin (S7).

XRD patterns of nature HA derived from fish scales and synthesized HA by using partially hydrolyzed keratin as a template is shown in Figure 1. The diffraction peaks appeared in these synthesized HA agree with those of the standard HA (JCPDS No. 09-0432) [7,8], which indicated that the HA crystals have been successfully synthesized. According to the Figure 1, peaks observed at 2θ degree of 26.1, 32.1, 33.0, 40.1, 47.0, 49.7 and 53.4 can be recognized to (002), (211), (300), (310), (222), (213) and (004) reflections of HA [26,27]. Broader peaks and lower peak resolution of HA (S6) from the fish scales indicate lower crystallinity and smaller size of HA nanocrystals than those of synthetic HA samples [28,29]. The XRD patterns of partially hydrolyzed keratin (S7) is also shown in Figure 1. The peak at 2θ degree of about 19 means the keratin used in this study is the β-sheet crystalline structure [30].

**Figure 2:**
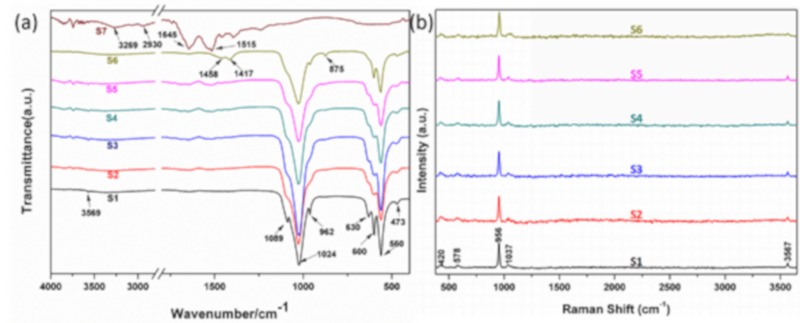
FTIR spectra (**a**) of synthesized HA samples (S1–S5), HA derived from grass carp scales and partially hydrolyzed keratin (S7) and Raman spectra (**b**) of synthesized HA samples (S1–S5) and HA derived from grass carp scales.

The FTIR spectra of nature HA derived from fish scales and synthesized HA by using partially hydrolyzed keratin as a template are shown in Figure 2. The absorption peaks at 1089, 1024 and 962 cm^−1^ can be attributed to υ1 and υ3 phosphate modes. The absorption peaks at 560 and 600 cm^−1^ are attributed to υ4 phosphate modes [31]. The peaks at 630 and 3569 cm^−1^ from S1 should be assigned to hydroxyl groups (OH^−^) [32]. With the increasing concentration of partially hydrolyzed keratin, the intensity of these two peaks becomes weak. We can hardly find OH signal at 3569 cm^−1^ from HA (S6) derived from fish scales. The antisymmetric stretching vibration of C–O (υ3) in the region of 1500–1400 cm^−1^ and υ2 vibration of CO_3_^2−^ at 875 cm^−1^ indicate that HA (S6) derived from fish scales contains CO_3_^2−^. S7 is the FTIR spectra of partially hydrolyzed keratin, the main peaks at 1515 and 1645 cm^−1^ are the peak of amino-group (N–H) and amide bond (CO–NH), respectively. Raman spectra of all HA samples are also shown in Figure 2. The characteristic peaks of PO_4_^3−^ at 420 and 578 cm^−1^ can be assigned to υ2 and υ4 mode, respectively [33]. The peaks at 1037cm^−1^ are corresponding to antisymmetrical stretching vibrations (υ3) of PO_4_^3−^ ion and peaks at 1070 cm^−1^ can be attributed to CO_3_^2−^ ions in HA crystal lattice [34,35]. The strongest symmetric stretch υ1 mode of PO_4_^3−^ is situated at 956 cm^−1^ and the O–H stretch can be found at ~3567 cm^−1^ [31]. All HA samples have an OH signal at ~3567 cm^−1^. However, the weak intensity of OH peak and strong signal of CO_3_^2−^ ions at 1070 cm^−1^ of HA (S6) derived from the fish scale is much lower than those of synthetic HA samples because some OH ions in apatite lattice have been substitute by CO_3_^2−^ ions.

**Figure 3:**
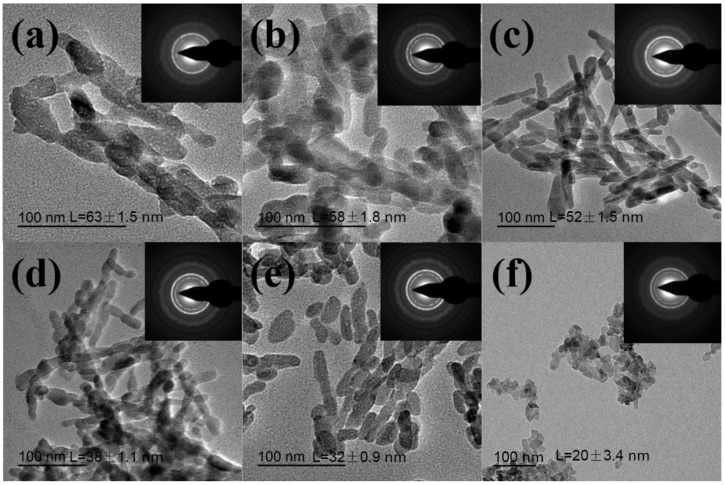
TEM and SEAD images of synthesized HA samples: (**a**–**e**) S1–S5 and HA derived from grass carp scales (**f**) S6.

In our previous study, we found the size of HA can be affected by the molecular weight of chitosan oligosaccharide [8]. However, the morphology and size are difficult to control under ~100 nm. Thus, inspired by recent findings on important roles of protein in biomineralization and natural structure of fish scales, we hypothesized that negatively charged segments of keratin could play an important role in regulating the mineralization process of HA crystals. TEM was used to characterize the morphology and size of synthesized and natural derived HA samples. Figure 3a shows that the length of synthesized HA nanocrystals without adding hydrolyzed keratin (S1) is about 63 ± 1.5 nm. With the increase of hydrolyzed keratin concentration, the length of HA nanocrystals decreased (Figure 3 b–e). The average length of S2, S3, S4, S5, and S6 is 58 ± 1.8 nm, 52 ± 1.5 nm, 38 ± 1.1 nm, 27 ± 0.9 nm and 20 ± 3.4 nm, respectively, indicating that the negatively charged segments on side chains of keratin play a key role in regulating the morphology and size of HA nanocrystals [36]. As previously known, partially hydrolyzed keratin contains many amino groups (–NH_2_), carboxyl groups (–COOH), mercapto groups (–SH) and even sulfonic groups (–OSO_3_H) and Ca^2+^ ions can bond with these functional groups. When the concentration of partially hydrolyzed keratin increases, the content of free Ca^2+^ in solution becomes lower, which causes the slower formation rate of HA crystals, eventually leading to the formation of smaller crystals [37]. Figure 3f shows that natural HA (S6) has an irregular shape and much smaller size than the other samples, which could be attributed to low crystallinity due to the high carbonate content. The selected area electron diffraction (SEAD) patterns show multi-crystalline electron diffraction concentrate rings attributed to (002), (102) and (211) crystallographic planes of HA [38]. In accordance with XRD and FTIR results, the size slightly decreased with the increased concentration of partially hydrolyzed keratin. The changes in the size of the nanocrystals were noticeable when 0.2 g of the partially hydrolyzed keratin was used. With the content of partially hydrolyzed keratin up to 0.6 g, the size of the nanocrystals ranged from 63 ± 1.5 nm to 27 ± 0.9nm. According to the results, the size of HA nanocrystals gradually decreased from about 63 ± 1.5 nm to 27 ± 0.9 nm as the content of partially hydrolyzed keratin increased from 0 to 0.6 g. Therefore, a new strategy for the synthesis of HA nanocrystals with controllable sizes by using partially hydrolyzed keratin as a templating agent was developed. In our opinion, the size and shape of the HA can influence their applications. Nanosized HA can be used as carriers of the drug, protein, and gene delivery due to their large surface area, improved sinterability and better bioactivity [39]. Hannig and Hannig also found these nano-sized apatite enamel crystallites may promote remineralization and physiological biofilm management of the tooth surface [40].

It is well known that synthetic HA is a bioactive material that is chemically similar to the mineral component of a bone. Indeed, the human bone is a natural composite comprising of nano-apatite rods (which are, 100 nm) arranged in lamellae and bound to collagen. Thus, synthetic nano-apatite is of interest as a biocompatible phase reinforcement in biomedical composites, for filling bulk bone defects and for coatings on metal implants [41]. Moreover, HA and other calcium phosphates (calcium deficient hydroxyapatite, CDHA) are also of interest as components in injectable bone cement; controlling particle properties (e.g., size and shape) is often used to modulate cement setting behavior. In this study, we chose the HA derived from carp scales as the control due to the following reasons. Firstly, HA from fish bone and scales has emerged as an alternative to substituting the synthetic and bovine HA in the last years since similar chemical properties have been achieved by simple and inexpensive methods [42]. Secondly, it has been demonstrated that fish sources are safe and present low risks of disease transmission [43]. Thirdly, fish are abundant in the environment, and the application of their byproducts is suitable for biomedical application as it would reduce environmental pollution and threats of biohazards to humans.

**Figure 4:**
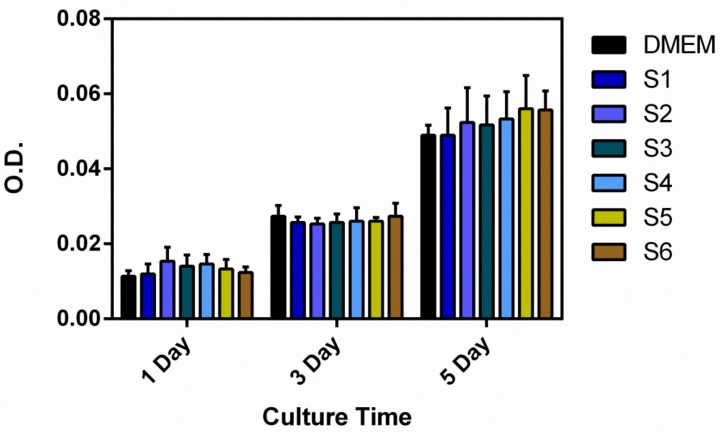
In vitro cytotoxicity of MG-63 cell lines after culturing in the synthesized HA samples (S1–S5) and HA derived from carp scales (S6) and DMEM for 1, 3 and 5 days.

**Figure 5:**
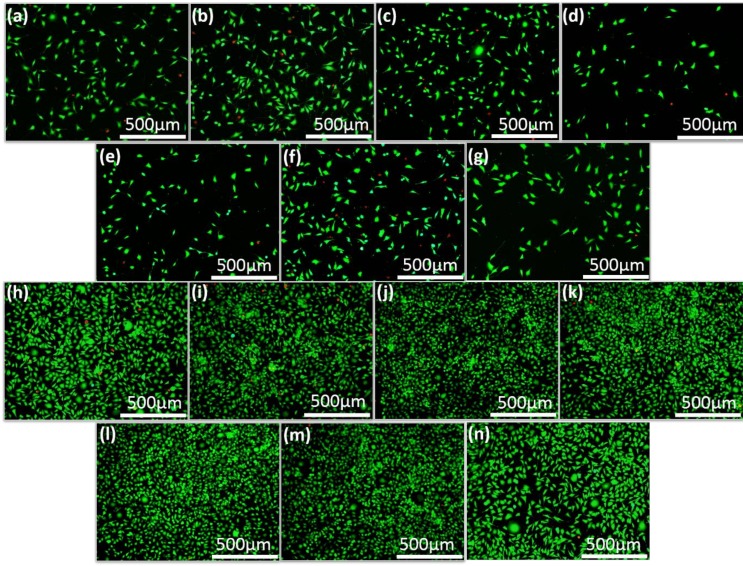
Live/Dead staining of MG-63 cells after cultured in HA samples and DMEM for 1 day (**a**–**g**) and 3 days (**h**–**n**). (**a**–**g**), (**h**–**n**) are S1–S6 samples and (**g**), (**n**) DMEM which acted as the control group.

In vitro cytocompatibility of synthesized and naturally derived HA was investigated using CCK-8 assay and Live/Dead fluoresce staining of MG-63 cell line. Proliferation results of MG-63 cells cultured in the HA samples for 1, 3 and 5 days are shown in Figure 4. Cell proliferation of all groups was similar, while there were obvious increases in the cell numbers after 3 and 5 days. This finding indicated that that MG-63 cells grew regularly on all synthesized HA nanocrystals. Further, it was clearly observed that all syntheses exhibited similar cell proliferation to the α-MEM control which indicated all synthesized HA nanocrystals have excellent cytocompatibility. The viability of MG-63 cells cultured in HA samples was also evaluated by Live/Dead staining after culturing for 1 and 3 days, as shown in Figure 5. Figure 5 also showed that the numbers of live cells increased on all samples after 3 days and the cells on all synthesized and α-MEM control proliferated to a confluence with very few dead cells. These results demonstrated that all synthesized HA nanocrystals have no cytotoxicity.

## 4. Conclusions

In the present study, a new strategy for synthesizing of HA nanocrystals with controllable sizes was developed by using partially hydrolyzed keratin as the templating agent. Results revealed that grain-like morphology of HA nanocrystals was obtained in the presence of partially hydrolyzed keratin. The concentration of partially hydrolyzed keratin in the process of the HA synthesis process influenced the HA crystal size. As the content of partially hydrolyzed keratin increased from 0 to 0.6 g, the size of HA nanocrystals gradually decreased from about 63 ± 1.5 nm to 27 ± 0.9 nm, which get close to crystal morphologies of natural apatites derived from fish scales. Results suggested that the partially hydrolyzed keratin play a positive role in the nucleation and growth of HA nanocrystals. In vitro, cytocompatibility assays showed that these synthesized HA nanocrystals enhanced MG-63 proliferation up to 5 days compared to natural derived HA. All these results indicated that prepared HA nanoparticles could be promising candidates for bone tissue engineering applications.

## Figures and Tables

**Table 1:** The formulae of synthesized HA samples (S1–S5) and HA derived from scales set as S6.

Sample	Ca(NO_3_)_2_·4H_2_O	(NH_4_)_2_HPO_4_	Partially Hydrolyzed Keratin
S1	9.54 g	3.20 g	—
S2	9.54 g	3.20 g	0.02 g
S3	9.54 g	3.20 g	0.1 g
S4	9.54 g	3.20 g	0.2 g
S5	9.54 g	3.20 g	0.6 g
S6	—	—	—
